# Enhanced catalysis of 
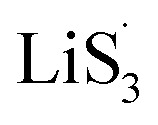
 radical-to-polysulfide interconversion *via* increased sulfur vacancies in lithium–sulfur batteries†

**DOI:** 10.1039/d2sc01353c

**Published:** 2022-05-10

**Authors:** Rui Xu, Hongan Tang, Yuanyuan Zhou, Fangzheng Wang, Hongrui Wang, Minhua Shao, Cunpu Li, Zidong Wei

**Affiliations:** The State Key Laboratory of Power Transmission Equipment & System Security and New Technology, Chongqing Key Laboratory of Chemical Process for Clean Energy and Resource Utilization, School of Chemistry and Chemical Engineering, Chongqing University Shazhengjie 174 Chongqing 400044 China lcp@cqu.edu.cn zdwei@cqu.edu.cn; Department of Chemical and Biological Engineering, The Hong Kong University of Science and Technology Clear Water Bay Kowloon Hong Kong

## Abstract

The practical application of lithium–sulfur (Li–S) batteries is seriously hindered by severe lithium polysulfide (LiPS) shuttling and sluggish electrochemical conversions. Herein, the Co_9_S_8_/MoS_2_ heterojunction as a model cathode host material is employed to discuss the performance improvement strategy and elucidate the catalytic mechanism. The introduction of sulfur vacancies can harmonize the chemisorption of the heterojunction component. Also, sulfur vacancies induce the generation of 
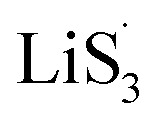
 radicals, which participate in a liquidus disproportionated reaction to reduce the accumulation of liquid LiPSs. To assess the conversion efficiency from liquid LiPSs to solid Li_2_S, a new descriptor calculated from basic cyclic voltammetry curves, nucleation transformation ratio, is proposed.

## Abstract 2

Sulfur vacancies can harmonize chemisorption and generate amounts of  radicals to boost the conversions of LiPSs.

## Graphical abstract

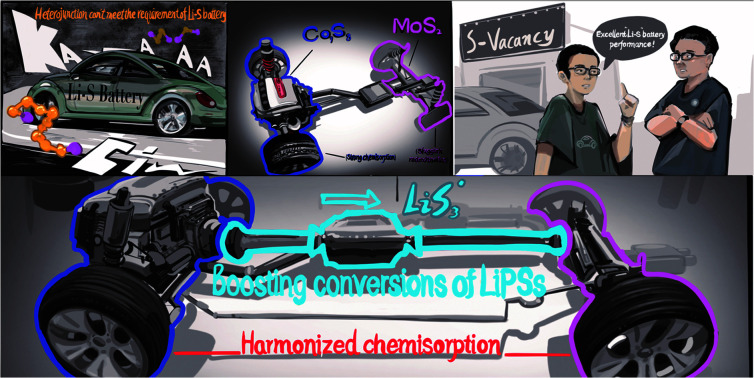

## Introduction

With the growing demands of high-energy-storage equipment, traditional lithium-ion batteries (LIBs) could fail to meet the increasing requirements for future renewable energies. Based on the ultrahigh theoretical specific capacity (1675 mA h g^−1^) of sulfur cathodes, lithium–sulfur (Li–S) batteries are one of the most promising and practical candidates for next-generation energy storage systems.^1–3^ Nevertheless, the commercialized application of Li–S batteries is hampered by their low practical capacity and poor cyclic stability, hindering their commercialization due to the sluggish redox kinetics for lithium polysulfide (Li_2_S_*x*_, 4 ≤ *x* ≤ 8) conversion and the notorious lithium polysulfide (LiPS) shuttling effect.^4–7^ To address the above problems, designed materials are reported based on physical confinement and chemisorption. To date, many host materials have been introduced for Li–S batteries.^8–13^ For example, porous carbon materials endow a porous structure to physically confine LiPS shuttling.^14^ Metal oxides have excellent chemisorption to alleviate LiPS shuttling.^8,11^ However, both of these strategies are passive methods to tie the LiPSs in interior or surrounding of the host materials. Driven by the concentration gradient of the soluble LiPSs, the notorious shuttling effect is still hard to avoid.^15,16^ Hence, LiPSs have gained wide interest to introduce catalysis into Li–S batteries, hoping this strategy will accelerate the conversion of LiPSs for sulfur reduction reactions and sulfur evolution reactions.^17–21^ Up to now, nitrides, carbides and sulfides have been widely recommended as catalysts.

**Scheme 1:**
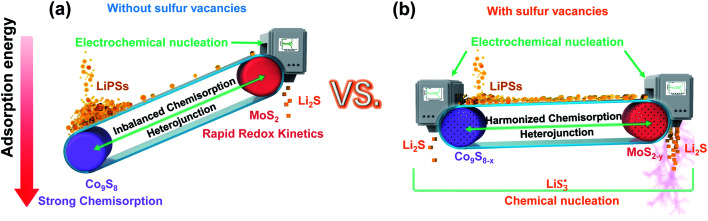
Catalytic mechanism by which sulfur-vacancy heterojunctions enhance the interconversion of LiPSs. (a) Conventional Co_9_S_8_/MoS_2_ heterojunction: Co_9_S_8_ was designed to adsorb liquid-phase LiPSs (orange-yellow liquid balls), while MoS_2_ was used to convert LiPSs to Li_2_S. However, liquid-phase LiPSs are strongly adsorbed by Co_9_S_8_ and therefore cannot be transferred to MoS_2_ to accomplish fast conversion. (b) By the introduction of sulfur vacancies, heterojunction materials can harmonize the chemisorption of components to uniformly adsorb LiPSs and produce abundant 
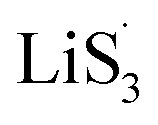
 radicals to facilitate the conversion of LiPSs to Li_2_S *via* a chemical nucleation route.

From recent studies, heterojunction host materials can endow composites with favorable physicochemical properties, one component to chemisorb LiPSs and the other to catalyze LiPS conversion. Although heterojunction host materials possess certain advantages to meet the requirements of Li–S batteries, they are trapped by the limitation of finite heterointerfaces and active sites, hardly achieving the expected rapid conversion of LiPSs.^20,22^ In addition, too strong chemisorption of LiPSs on one component will block soluble LiPSs from moving to the other component, where further electrochemical conversions of LiPSs take place (Scheme 1(a)). Moreover, on the liquid–solid interface, it is not easy for Li_2_S_4_ species to gain two electrons and convert themselves to solid-phase Li_2_S_2_ in the whole electrochemical conversion process.^23–26^ Thus, it is difficult to obtain suitable chemisorption and accelerate the conversions of LiPSs for Li–S batteries.^15,25,27,28^ Of note, defect engineering is often used to modulate the properties of materials without introducing other elements. In the case of the Co_9_S_8_/MoS_2_ heterojunction, sulfur vacancies can be naturally thought to tailor the chemisorption and catalytic properties.^29^ However, the catalytic mechanisms are usually discussed but do not expound clearly.

Based on the abovementioned discussion, reducing the accumulation of liquid LiPSs is crucial for the performance of Li–S batteries. We chose Co_9_S_8_/MoS_2_ heterojunction composites with sulfur vacancies as a template sulfur host to demonstrate how sulfur vacancies modulate the behavior of the heterojunction composites with respect to chemisorption and LiPS conversions (Scheme 1(b)). With the introduction of sulfur vacancies, a large number of free radicals 
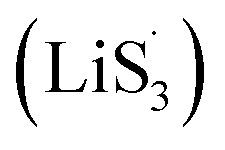
 are generated on the sites of sulfur vacancies to promote the nucleation of Li_2_S *via* a liquidus disproportionated reaction. In addition, the harmonized chemisorption of heterojunctions can speed up LiPS transport between the two components and then maximize the catalytic effect to achieve rapid LiPS conversion. Furthermore, to assess the accumulation behavior of the liquid LiPSs, a new quantitative descriptor, Nucleation Transformation Ratio (NTR), is proposed. NTR is defined as the ratio of the die-out amount to the formation amount of liquid LiPSs, and can be calculated from basic cyclic voltammetry curves. Therefore, the transformation efficiency of S-related species in Li–S battery can be reflected quantitatively.

## Experimental

### Chemicals

Methyl-2-pyrrolidone (NMP, 99.5 wt%), polyvinylidene fluoride (PVDF, 99.5 wt%), elemental sulfur (99.5 wt%), lithium sulfide (Li_2_S, 99.98 wt%), 1,3-dioxolane (DOL, 99.8 wt%), 1,2-dimethoxyethane (DME, 99.5 wt%), tetraglyme (99 wt%), lithium bis (trifluoromethanesulfonyl) imide (LiTFSI, 99.95 wt%), lithium nitrate (LiNO_3_, 99.99 wt%), cobalt chloride hexahydrate (CoCl_2_·6H_2_O), potassium permanganate (KMnO_4_, 99.5 wt%), graphite powder, ammonium tetrathiomolybdate ((NH_4_)_2_MoS_4_, 99.95 wt%), reduced graphene oxide (rGO, chemically reduced) were purchased from Sigma-Aldrich. Graphene oxide (GO, >99 wt%) was purchased from Aladdin reagent. Hydrochloric acid (HCl, 37 wt%), sodium nitrate (NaNO_3_, 99 wt%), hydrogen peroxide (H_2_O_2_, 30 wt%) were purchased from Chuandong Reagent. Super P carbon (99.5 wt%) from Timcal were used as received. The carbon paper (HCP120, thickness *∼*0.21 mm) was purchased from Shanghai HESEN Co., Ltd.

### Preparation of Co_9_S_8_/MoS_2_-rGO composite

The Co_9_S_8_/MoS_2_-rGO composites were synthesized through a simple hydrothermal synthesis and subsequent pyrolysis process. Briefly, 100 mg of graphene oxide (GO) powder was mixed with 45 mL deionized water, then ultrasonicated for 3 h to obtain the GO suspension. CoCl_2_·6H_2_O (5 mL; 8 mg mL^−1^) aqueous solution was then dropwise added into the prepared GO suspension under vigorous sonication for 30 min. Then, 35 mg (NH_4_)_2_MoS_4_ dispersed in 15 mL of deionized water similarly was added into the above-mentioned suspension drop by drop. The obtained suspension was transferred into a 100 mL Teflon-lined stainless-steel autoclave. The autoclave was moved to a oven to heat at 200 °C for 24 h. After annealing to room temperature, the solid product was collected by centrifugation and then washed several times with deionized water. The precursor was then freeze–dried at −70 °C. Finally, the precursor was annealed at 700 °C in N_2_ atmosphere using a heating rate of 2 °C min^−1^ to obtain the CMG-L composites.

### Preparation of CMG composites

The CMG-M and CMG-H composites with sulfur deficiency were formed by heating the CMG-L products in a H_2_/N_2_ (10%/90%) mixed gas. The reaction temperatures were chosen for 400 °C and 700 °C. All the composites are collectively called CMG.

### Preparation of sulfur composite cathode materials

The above CMG composite powders and sublimed sulfur with a weight ratio of 3 : 7 were mixed and ground. Then the mixture was heated to 155 °C and kept for 12 h in a tube furnace under an N_2_ atmosphere to obtain the sulfur composite cathode materials.

### Materials characterization

The prepared materials was characterized with various morphology and spectroscopy methods. The scanning electron microscopy (SEM) images were collected with a JEOL JSM-7800F. The transmission electron microscope (TEM) and high resolution transmission electron microscopy (HR-TEM) observations were performed with Tecnai G2F20 TWIN and JEM-2100F, respectively. X-ray diffraction (XRD) were performed with Rigaku D/max 2200 pc diffractometer under 40 kV and 40 mA with monochromatic Cu (K_α_) radiation (*λ* = 1.54 Å). Raman spectra, ultraviolet-visible (UV-Vis) spectra, and X-ray photoelectron spectroscopy (XPS) were collected with Labram HR800 (HORIBA Jobin Yvon)), Lambda 750 spectrophotometer (PerkinElmer), and ESCALAB250Xi (Thermo Scientific instrument) with Al (K_α_) (1486.6 eV) radiation, respectively. Thermo gravimetric analysis (TGA) was carried out by a Mettler Toledo TGA in the temperature range of 25 to 600 °C at a heating rate of 10 °C min^−1^ under N_2_ atmosphere. The pore structure and distribution was analyzed with Brunauer–Emmett–Teller (BET) method using a Micromeritics ASAP 2460 BET analyzer.

### Visualized adsorption test

10 mg of rGO, CMG-L, CMG-M and CMG-H were added into 2000 μL Li_2_S_6_ solution with ultrasonic dispersion for 1 min, followed by static adsorption in Ar-filled glovebox. The used Li_2_S_6_ solution was prepared by mixing lithium sulfide and element sulfur into the electrolyte based on the mixing solvent of DOL/DME with a volume ratio of 1 : 1 to form saturated 1 mM Li_2_S_6_ solution. All the operations were performed in an Ar-filled glovebox. After absorption for 12 h, the supernatant solution was poured into the cuvette for UV-Vis spectrum test on a Lambda 750 spectrometer.

### Li_2_S nucleation tests

Elemental sulfur and Li_2_S was vigorous mixed for 24 h with a molar ratio of 7 : 1 in tetraglyme to obtain the 0.20 mol L^−1^ Li_2_S_8_ electrolyte. Carbon papers were punched into 12 mm circle disks to load CMG composites. The loading was controlled to be 1.0 mg cm^−2^. Lithium foils and the obtained CMG loaded carbon paper were used as the anode and cathode, respectively. LIR2032 coin cells was assembled with Celgard 2400 separator. The cathode was firstly be wetted with the previously prepared Li_2_S_8_ electrolyte, and the other 20 μL of LiTFSI (1.0 mol L^−1^) was added into the LIR2032 coin cell. These cells should be firstly galvanostatically discharged to 2.06 V, and then switched to 2.05 V potentiostatically test until the current below 10^−5^ A. These procedure to guarantee the fully precipitation of Li_2_S.^30^

### Symmetric cell assembly and measurement

Typically, Co_9_S_8_/MoS_2_-rGO or CMG composites were mixed with PVDF and carbon black (with a mass ratio of 3 : 1 : 1) in NMP solvent. Then the mixtures were uniformly coated onto carbon papers. The average mass loading of electrodes were controlled at about 1 mg cm^−2^. Two identical electrodes as working and counter electrodes were assembled into a standard LIR2032 cell with a Celgard 2400 separator in an Ar-filled glovebox. 0.1 mol L^−1^ Li_2_S_6_ in 40.0 μL of DOL/DME (with a volume ratio of 1 : 1) was used as electrolyte, which also contained 1.0 M LiTFSI. Cyclic voltammetry (CV) tests were carried out on an electrochemical workstation (CHI660D, Shanghai Chenhua) at a scan rate of 1.0 mV s^−1^. The voltage range of CV measurement was −0.8 to 0.8 V. The symmetric cell with Li_2_S_6_-free electrolyte was also tested as a reference.

### Electrochemical tests

Electrochemical tests of these electrode materials were performed using coin-type (LIR2032) cells. The cells were assembled with the prepared sulfur composite cathodes (active material : carbon black : PVDF = 8 : 1 : 1), lithium anodes, electrolyte and Celgard 2400 in an argon filled glove box with extremely low H_2_O and O_2_ concentrations (<1.0 ppm). For compatibility of sulfur cathode and lithium anode, DOL/DME formulation which possess moderate solvating capability for LiPSs was chosen as the solvent.^31^ The used electrolyte was 1 M LiTFSI dissolved in a mixed solvent of DOL/DME (volume ratio of 1 : 1). For each composite cathode, the average areal loading was around 3.2 mg cm^−2^ and sulfur content was about 68 wt%, with an electrolyte volume of 50 μL in full cells (the diameter of cathode is 12 mm, the electrolyte-to-sulfur ratio was 13.8 μL mg^−1^). The mass of the corresponding carbon paper was measured to be around 17.7 mg. The galvanostatic charge–discharge (GCD) tests were conducted on LAND CT2001A between 1.6–2.8 V (*vs.* Li/Li^+^). Galvanostatic intermittent titration technique (GITT) tests were performed on Land battery test system with discharging current of 167.2 mA g^−1^ for 0.5 h and resting for 2.5 h. The specific capacity and current rates (1C = 1672 mA h g^−1^) were calculated on the basic of the sulfur weight in the cathode. The CV tests were performed with an electrochemical workstation CHI660D with the cut-off voltage of 1.6–2.8 V. To evaluate the conductivities of the samples, CMG samples with different sulfur vacancies were collected after annealing process. Subsequently, under the press of 10 MPa, the obtained powders (about 100 mg) were compacted *via* an infrared spectroscopy tablet mould to obtain corresponding compact sheets. It should note that the diameter of the tablet mould is 12 mm and the sheet thickness was measured with an micrometer. The obtained compact sheets were assembled in LIR2032 coin-type cells without electrolyte for conductivity tests. Electrochemical impedance spectroscopy (EIS) data were recorded by applying a sine wave with 5 mV fluctuation from a frequency range of 100 mHz to 10^3^ kHz (Princeton 1260A impedance analyser). Besides, the conductivity formula is as follows:
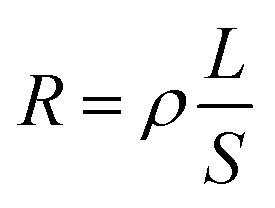

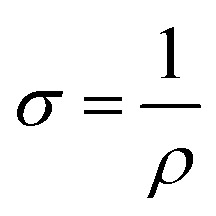
where *R* is the resistance, *ρ* is the specific resistance, *L* is the thickness of sheet, *S* is the area of the sheet, and *σ* is the electronic conductivity, respectively. All the electrochemical tests were performed at room temperature.

### Theoretical calculation

Vienna *ab initio* simulation package (VASP) software was used to perform the density functional theory (DFT) theoretical calculations. The DFT+U calculation was implemented with the Perdew–Burke–Ernzerhof (PBE) functional within the generalized gradient approximation (GGA).^32,33^ To assess the adsorption behavior of LiPS and base materials, a 15 Å vacuum layers was set in the *z*-direction to avoid inter-layer interactions. For all the structural optimization processes, the bottom three layers were fixed and the other atoms were fully relaxed to reach a thermodynamical stable state. The cut-off energy was set to be 500 eV. 3 × 2 × 1 and 2 × 2 × 1 *k*-point sampling was provided for Co_9_S_8_ and MoS_2_, respectively. The convergence criterion was set to be 0.02 eV Å^−1^ for force on each atom and 10^−5^ eV for total energies during the geometry optimization calculations. The adsorption energy (Δ*E*_ads_) of the species on base surface was determined by the following equation:^33^Δ*E*_ads_ = *E*_ads/base_ − *E*_ads_ − *E*_base_where *E*_ads/base_, *E*_ads_ and *E*_base_ are the total energy of the adsorbed systems, the isolated Li_2_S_6_, and base materials, respectively.

## Results and discussion

**Fig. 1:**
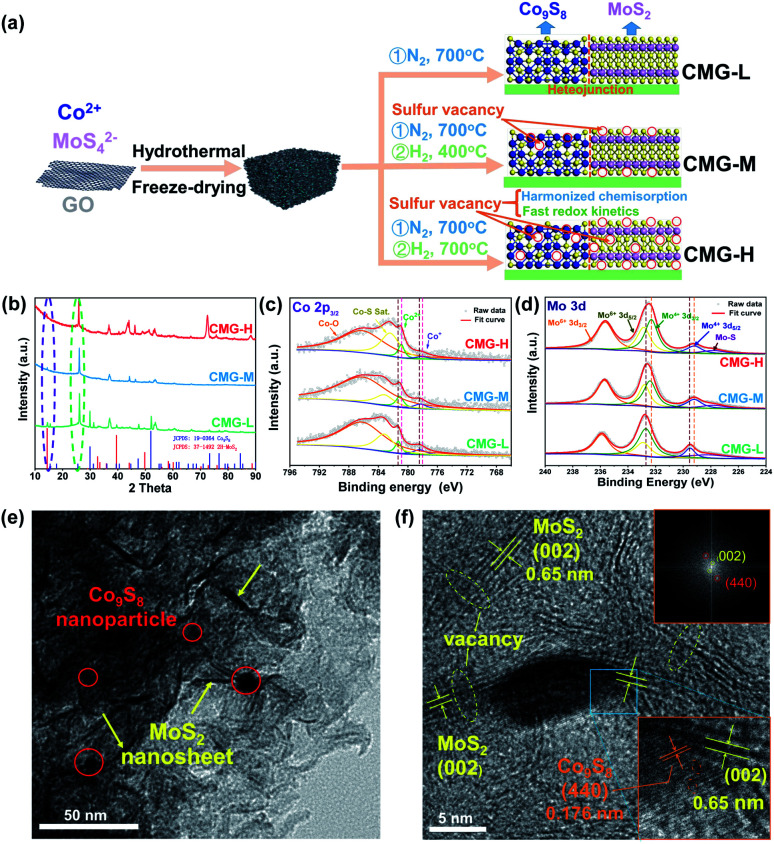
(a) Schematic of the synthesis of the CMG composite; (b) XRD spectra of CMG-L, CMG-M and CMG-H; high-resolution XPS spectra of Co 2p (c) and Mo 3d (d) for CMG-L, CMG-M and CMG-H; TEM (e) and selected area electron diffraction (SAED) images (f) of CMG-H.

From previous studies, Co_9_S_8_ endows strong chemisorption to alleviate LiPS shuttling.^34^ In addition, MoS_2_ possesses weak chemisorption yet can significantly enhance the LiPSs conversion kinetics.^35^ Herein, we designed a sulfur-vacancy heterojunction material based on Co_9_S_8_ and MoS_2_ loaded with rGO to explore the promotion mechanism. The synthetic process of CMG with different sulfur vacancy densities was prepared following a hydrothermal and annealing method in different atmospheres, as illustrated in Fig. 1(a). Consequently, we defined the obtained CMG with a low sulfur vacancy as CMG-L, a middle sulfur vacancy as CMG-M and a high sulfur vacancy as CMG-H. The characteristic peaks in XRD spectra revealed the presence of two crystal structures: MoS_2_ and Co_9_S_8_ (Fig. S1†). XPS was used to confirm the heterojunction structure of CMG-L and MoS_2_-rGO (Fig. S2†). The Mo 3d binding energy (BE) indicates that the BE peaks at 228.9 eV and 231.9 eV are pointed to Mo^4+^ for CMG-L. Compared with MoS_2_-rGO, the 0.17 eV negative shift of the Mo^4+^ BE peak of CMG-L indicates the strong interaction and electron transfer between MoS_2_ and Co_9_S_8_. This observation implies that the heterojunction structure of Co_9_S_8_ and MoS_2_ were successfully obtained.^36,37^

Based on previous research, the annealing atmosphere and temperatures were regulated, resulting in different sulfur vacancy concentrations.^34,35,38^ SEM images of the CMG composites revealed a porous, sponge-like morphology consisting of countless erect sheets with a lacunose interconnected structure (Fig. S3†). Moreover, with increasing sulfur vacancy concentration, CMG-H exhibited the largest specific surface area *via* N_2_ adoption–desorption isotherms, which displayed good exposure of sulfur vacancies on the catalyst surface for LiPS interconversions (Fig. S4†).

As shown in Fig. 1(b), the XRD peaks at the same location were in good agreement with Co_9_S_8_ and MoS_2_ for the CMG samples with different sulfur vacancy concentrations. With the increasing of the sulfur vacancy concentration, the negative shift of the 2*θ* degree peak at ∼14° (MoS_2_ (002)) and ∼26° (Co_9_S_8_ (220)) in the expanded view (Fig. S5†) indicated lattice expansions, which were mostly caused by the removal of sulfur by hydrogen.^34,35^ In addition, the XPS binding energy of Mo and Co binding energy shifted to a lower position, which also proved the partial reduction of Co_9_S_8_ and MoS_2_ (Fig. 1(c and d)).^38^ The sulfur vacancy concentrations can be calculated from the S ratio loss (from 0.09% to 0.51%) from Table S1,† which is consistent with the above analyzed fine spectra.

The sulfur-vacancy heterojunction material CMG-H was further investigated by TEM. As shown in Fig. 1(e) and S6,† the heterojunction is anchored on the reduced graphene oxide. Furthermore, Co_9_S_8_ exists in the form of nanoparticles, and MoS_2_ exists in the form of nanosheets. HR-TEM images of the Co_9_S_8_/MoS_2_ heterogeneous interface are shown in Fig. 1(f). Lattice fringes with spacings of 0.174 nm and 0.65 nm were indexed to the (440) plane of Co_9_S_8_ and the (002) plane of MoS_2_, respectively. The corresponding SEAD pattern (inset of Fig. 1(f)) also consistent with both planes. Moreover, the interface between Co_9_S_8_ (440) and MoS_2_ (002) can be observed, and discontinuous (002) and (440) facet crystal fringes also emerged on account of the presence of abundant sulfur vacancies.

**Fig. 2:**
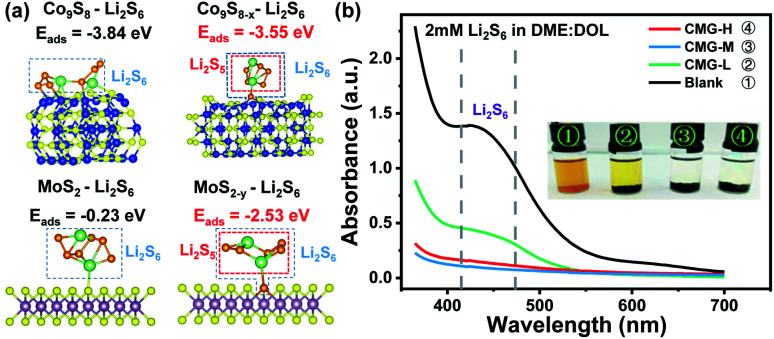
(a) Calculated adsorption energies (Δ*E*_ads_) of Li_2_S_6_ on the (002) planes of MoS_2_ and MoS_2−*y*_ crystals as well as (220) planes of Co_9_S_8_ and Co_9_S_8−*x*_ crystals; (b) the adsorption abilities of three host materials with Li_2_S_6_ as the representative lithium polysulfides.

It is well known that the chemisorption ability plays a significant role in inhibiting LiPS shuttling during cycling. Hence, to investigate the influence of different sulfur vacancy concentrations on chemisorption ability, first-principal calculations based on density functional theory (DFT) were used to probe the chemical adsorption energies between the components of the heterojunction and the representative soluble LiPS – Li_2_S_6_. As shown in Fig. 2(a), the adsorption energy between Li_2_S_6_ and Co_9_S_8_ (Δ*E*_ads_ = −3.84 eV) is much stronger than that between Li_2_S_6_ and MoS_2_ (Δ*E*_ads_ = −0.23 eV). From the view of thermodynamics, the results demonstrated that MoS_2_ is relatively easily adsorbed by Li_2_S_6_; and Co_9_S_8_ is more favorable for Li_2_S_6_ desorption. After the introduction of sulfur vacancies, Co_9_S_8−*x*_ presented a weaker Δ*E*_ads_ (−3.55 eV) than Co_9_S_8_ (−3.84 eV). However, the plane of MoS_2−*y*_ (002) showed a distinctly stronger Δ*E*_ads_ (−2.53 eV) than that of MoS_2_ (−0.23 eV) for Li_2_S_6_ adsorption. In this regard, the Δ*E*_ads_ difference for Co_9_S_8−*x*_ and MoS_2−*y*_ was significantly reduced after the introduction of sulfur vacancies. The chemisorptions strength between the heterojunction components are therefore harmonized. It is interesting that for Co_9_S_8−*x*_ and MoS_2−*y*_, one of the S atoms in the chemisorbed Li_2_S_6_ will be unsymmetrical adsorbed to the sulfur vacancy, then leave a relatively reactive Li_2_S_5_ species, which may regulate the subsequent LiPS interconversions.

The harmonized chemisorption behavior was further verified from the visualized adsorption tests. Visualized adsorption tests were adopted *via* heterojunction materials soaked in Li_2_S_6_ solution (inset of Fig. 2(b)). CMG-L can slightly lighten the color of the Li_2_S_6_ solution. The CMG-M and CMG-H composites can fully decolor the Li_2_S_6_ solution, suggesting their superior adsorption abilities for LiPSs. UV-Vis absorption tests provided quantitative comparisons to the chemisorption abilities of the materials. From Fig. 2(b), compared with the blank group and the CMG-L material, the CMG-M and CMG-H materials rarely showed no adsorption peaks in the 400–500 nm region for Li_2_S_6_.^4,39^ Also, as expected from the DFT calculation, the absorbance of CMG-M material with moderate sulfur vacancies exhibited the lowest Li_2_S_6_ signal, which can be attributed to the harmonized chemisorption – these exists an adsorption extremum for the heterojunction materials, because the different adsorption-energy shift directions for Co_9_S_8−*x*_ and MoS_2−*y*_.

**Fig. 3:**
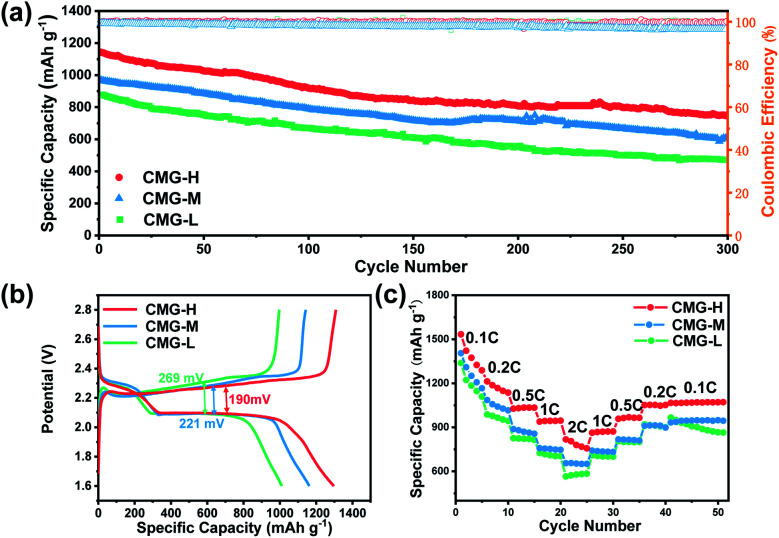
(a) Long-term cycling performances of CMG-L, CMG-M and CMG-H at 0.5C. The sulfur loading was 3.2 mg cm^−2^; (b) galvanostatic charge/discharge profiles of CMG-L, CMG-M and CMG-H at 0.2C; (c) rate capability performance of CMG-L, CMG-M and CMG-H.

Commonly, a higher chemisorption ability corresponds to a better electrochemical performance, owing to the suppression of LiPS shuttling. However, although the chemisorption ability does not monotonously increase with sulfur vacancies, the electrochemical and battery performances nevertheless increase with sulfur vacancies. Sulfur composite cathode materials were made by the melt diffusion method (Fig. S7†) for CMG-L, CMG-M, and CMG-H. The sulfur loading was controlled to ∼3.2 mg cm^−2^, and the proportion was around 68% in the sulfur composite cathodes (Fig. S7†). Full cells were fabricated using lithium metal as the anode. From Fig. 3(a), full cells using the CMG-H electrode delivered the highest initial capacity (1129 mA h g^−1^) and lowest capacity decay rate (0.108% per cycle, after 300 cycles) at 0.5C. Of note, the high sulfur-vacancy composite cathodes CMG-H battery delivered the best charge/discharge specific capacity (1308 mA h g^−1^). Moreover, the plateau gap of CMG-H (190 mV) between charge/discharge is much smaller than that of CMG-L (221 mV) and CMG-M (269 mV), indicating that CMG-H possesses better kinetics during the charge/discharge process (Fig. 3(b)). In addition, as shown in Fig. 3(c), the CMG-H batteries exhibited the best rate performances, delivering discharge capacities of 1534, 1215, 1024, 938, and 816 mA h g^−1^ at rates of 0.1C, 0.2C, 0.5C, 1C, and 2C, respectively. When the rate gradually shifted back from 2C to 0.1C, the CMG-H battery also exhibited the best reversibility and excellent stability. It was extraordinary that CMG-M, with the strongest chemisorption, cannot express the best charge/discharge performance.

The electron-transport ability difference could be one contribution factor to this phenomenon. It can be found that the electronic conductivities of CMG gradually improved with the increase of sulfur vacancies, which gives rise to fast transport of electrons (Fig. S8†).^30,40^ Besides, EIS measurements also registered the smallest charge transfer resistance (the size of the high-frequency semicircle in the Nyquist plot) for the CMG-H electrode (Fig. S9 and Table S2†). These results suggested that the CMG-H electrode can possess better conductivity ability, meaning that the adsorbed LiPSs can gain electrons more easily to convert to solid phase Li_2_S. However, except for the conductivity difference, the reaction kinetics should also be discussed to understand the difference between CMG-M and CMG-H.

**Fig. 4:**
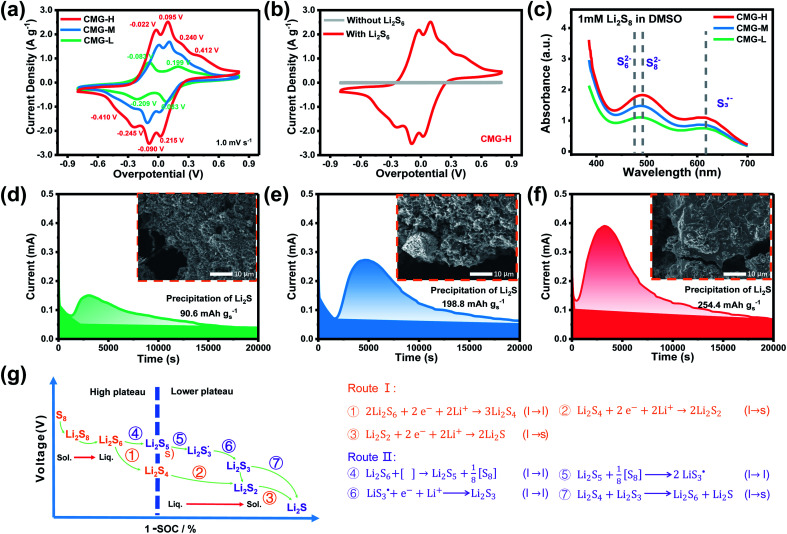
(a) Cyclic voltammograms of symmetric cells at 1 mV s^−1^; (b) cyclic voltammograms of symmetric cells with and without Li_2_S_6_ solution; (c) the variation in UV absorbance with increasing sulfur vacancy concentration (d–f) potentiostatic discharging curves of Li_2_S_8_/tetraglyme solution at 2.05 V and the corresponding SEM images after Li_2_S deposition; (g) proposed sulfur reduction reaction routes for the Li–S battery with the sulfur-vacancy heterojunction material.

With the increase of sulfur vacancies, faster reaction kinetics could be beneficial to improve the electrochemical performance of the battery. Therefore, it is worth further to determine the reason that the slightly weaker chemisorption of the high sulfur-vacancy heterojunction material CMG-H exhibited better electro-chemical performance than the low sulfur-vacancy material. Hence, it is meaningful to evaluate the role of sulfur vacancies on the interconversion reactions of LiPSs. CV tests were performed within a voltage window of −0.8 V to 0.8 V for symmetrical cells assembled by heterojunction materials with different sulfur vacancies (Fig. 4(a and b)).^41^ The CMG-L materials exhibited two pairs of broad peaks at −0.209/0.199 V and −0.083/0.083 V, which can be assigned to the conversion between Li_2_S_6_ and Li_2_S_*x*_ (*x* < 6), as well as the conversion between S_8_ and Li_2_S_6_, respectively.^42^ Moreover, as the sulfur vacancies increased, the peak current densities increased and the voltage hysteresis between the cathodic peaks and anodic peaks gradually decreased, indicated that the sulfur vacancies could dynamically accelerate the electrochemical reactions of LiPSs.^43^ The appearance of staged peaks at negative potentials, which correspond to the relatively short-chain polysulfide anion (S^2−^_4_) or free radical 
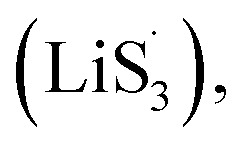
 is worth comprehensively discussing to understand the role of sulfur vacancies.^18,20,34,35,39,41,44^ The appearance of sulfur radicals 
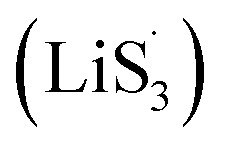
 promoted by sulfur vacancies was tested by UV-Vis spectroscopy in a 1 mM Li_2_S_8_ solution. Li_2_S_8_ solution was used to simulate the active sulfur source, and dimethyl sulfoxide (DMSO) was used as the solvent because it could stabilize 
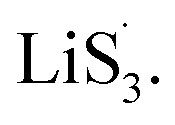
 From Fig. 4(c), it is obvious that more sulfur vacancies correspond to more sulfur radicals 
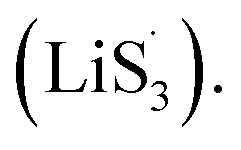
 Sulfur radical was believed to be generated from Li_2_S_5_, which was produced from the sulfur-vacancy sites and Li_2_S_6_ (Fig. 2(a)). The formed Li_2_S_5_ is extremely unstable and easily converts to other LiPS intermediates, such as 
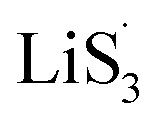
 radicals.

It is known that 
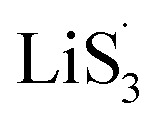
 could accelerate the interconversion of LiPSs by providing additional chemical pathways, particularly for Li_2_S deposition.^20,39,45^ Therefore, to further investigate the role of sulfur vacancies, Li_2_S precipitation experiments were carried out with the above three host materials as electrodes (Fig. 4(d–f)). Galvanostatic discharge was conducted to 2.06 V, and then the voltage was kept at 2.05 V until the current was below 10^−5^ A.^46^ Nucleation experiments show that the capacity of precipitated Li_2_S on CMG-H (254.4 mA h g_s_^−1^) is much higher than those of the two other heterojunction materials (90.6 mA h g_s_^−1^ and 198.8 mA h g_s_^−1^).

As shown in the insets of Fig. 4(d–f), after the deposition of Li_2_S, the surface of CMG-H is smoother and more uniform than the two other materials. It is interesting that the Li_2_S peak appeared much earlier for CMG-H than the others during the galvanostatic discharge process. This observation implies that the reduction reactions of LiPSs to Li_2_S occur much easier in CMG-H cathode, which can contribute to the detected electric current earlier. We named this current generated from all the S-related species involved reactions as the hybrid current. The earlier occurrence of the Li_2_S deposition current suggests that the liquid LiPSs will not accumulate but fastly be reduced to solid Li_2_S. Therefore, in the hybrid current, a greater contribution from LiPSs conversion to Li_2_S will decrease LiPSs shuttling to increase the stability of Li–S battery.

Based on the above discussion of the relationship of chemisorption and sulfur vacancies, the sulfur vacancies in the heterojunction could not only to harmonize the chemisorption, but also to adjust the redox kinetics process of LiPSs as follows. As illustrated in Fig. 4(g), harmonization of the chemisorption could hold back the LiPS shuttling. In the meantime, without the participation of 
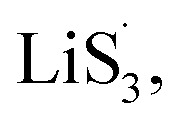
 only the conventional electrochemical nucleation route (route I) can accomplish liquid–solid transformation. As the electrochemical requirement is crucial for liquid-phase Li_2_S_4_ to gain two electrons on the liquid–solid interface of the cathode to be converted to solid-phase Li_2_S_2_.^43,47^ Li_2_S and Li_2_S_2_ deposition is controlled by the sparsely distributed nucleation sites on the liquid–solid interface of the cathode. In comparison, in addition to the route I, sulfur vacancies can promote the formation of sulfur radicals 
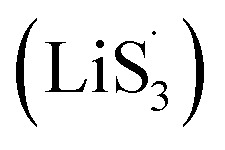
 to accelerate liquid-phase conversion (chemical nucleation route, route II). Taking the discharge process as an example, sulfur vacancies chemisorb Li_2_S_6_ to form reactive Li_2_S_5_, and then Li_2_S_5_ reacts with solid S_8_ to form 
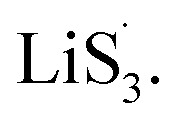
^47–50^ Liquid-phase 
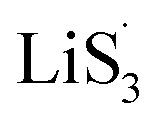
 gains one electron and one Li^+^ to convert to liquidus Li_2_S_3_ (reaction ⑦). Then, Li_2_S_3_ may react with Li_2_S_4_ to convert to Li_2_S_6_ and solid-phase Li_2_S *via* a spontaneous liquid-phase disproportionated reaction (Δ*H* = −1.31 eV), simultaneously achieving the formation of homogeneous nucleation sites.

**Fig. 5:**
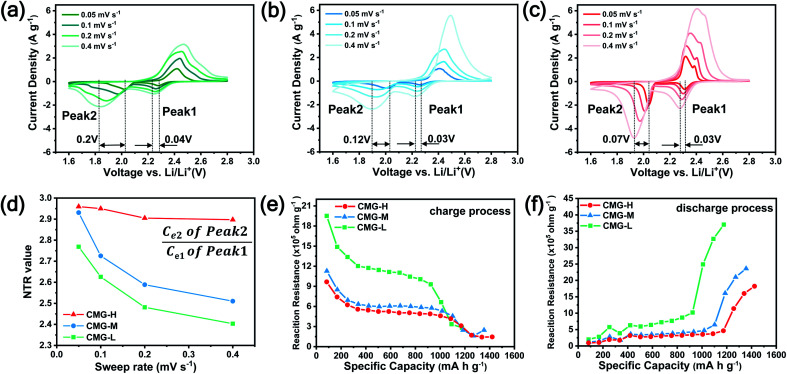
CV curves within the voltage range of 1.6–2.8 V at different sweep rates: (a) CMG-L. (b) CMG-M. (c) CMG-H. (d) The NTR values of CMG-L, CMG-M and CMG-H at different sweep rates; *in situ* reaction resistances during discharge (e) and charge (f) processes of CMG-L, CMG-M and CMG-H during GITT measurement.

As the function of 
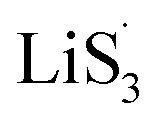
 is crucial for the deposition of Li_2_S and the formation of the hybrid current, the redox kinetics of heterojunctions with different sulfur vacancies were further studied by CV (Fig. 5(a–c)).^51^ Two cathodic peaks, located at 2.20–2.30 V (peak 1) and 1.80–2.05 V (peak 2) should correspond to the reduction of sulfur into high-order LiPSs (Li_2_S_*x*_, 4 ≤ *x* ≤ 8) and further LiPSs to low-order Li_2_S_2_/Li_2_S.^24–26,52^ Simultaneously, the broad anodic peaks should be assigned to the oxidation of Li_2_S_2_/Li_2_S to Li_2_S_6_/S_8_*via* the formation of intermediate LiPSs.^53^ Compared with the CMG-L and CMG-M electrodes, the CMG-H electrode showed distinguished, smaller polarization, demonstrating that peak 1 and peak 2 of CMG-H shifted 0.03 V and 0.07 V, respectively, with the scan rate increase (Fig. 5(a–c)).

High-efficiency Li_2_S deposition was quantitatively analysed *via* CV curves. The discharge currents for peak 1 and peak 2 were integrated with time as the quantities of charge for the corresponding reactions. Herein, the quantity of electron transfer was defined for the three heterojunctions based on the following equation:1
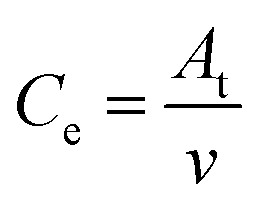
where *C*_e_ is the amount of electron transfer per gram (A s), *A*_t_ is the integral area (A V) of peak 1 and peak 2, and *v* is the scan rate (V s^−1^). In addition to the above discussed polarization behaviors, the calculated *C*_e_ can provide quantitative information for the interconversions of the LiPSs.

As illustrated in Table 1, 1 mol of solid-phase S_8_ molecules will obtain 4 mol of electrons to form 2 mol of liquid-phase Li_2_S_4_, which corresponds to peak 1 (reaction (1) in Table 1). These Li_2_S_4_ molecules will gain 12 mol of electrons to yield 8 mol of Li_2_S (peak 2, reaction (2) in Table 1). Therefore, a quantitative descriptor, named as Nucleation Transformation Ratio (NTR), to assess the kinetics behaviors of the cathode reactions can be defined:2
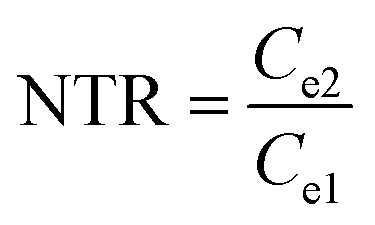
where *C*_e1_ and *C*_e2_ are the calculated *C*_e_s for peak 1 and peak 2, respectively. NTR can reflect the ratio of the reacted Li_2_S_4_ in peak 2 (die-out amount of LiPSs) to the produced Li_2_S_4_ in peak 1 (formation amount of LiPSs, more details in Fig. S10†). Based on the discussions in Table 1, the ideal NTR should be 3. The calculated NTR is closer to 3, the closer the conversion of Li_2_S_4_ to Li_2_S is to the ideal conditions. All the NTR values for CMG composites at different scan rates were calculated and are illustrated in Fig. 5(d) and Table S3.† At a lower scan rate (0.05 mV s^−1^), all the NTR values for the three samples were close to 3, while CMG-H exhibited the largest NTR = 2.96. As the scan rate increased, the voltage rapidly passed through the reactive window. Under high scan rate conditions, the produced Li_2_S_4_ from peak 1 cannot rapidly be converted to Li_2_S (peak 2) when the Li_2_S nucleation is blunt. As shown in Fig. 5(d), although the NTR for CMG-L and CMG-M decreased rapidly (2.40 for CMG-L and 2.51 for CMG-M at 0.4 mV s^−1^, respectively), the NTR for CMG-H remains close to 3. Meanwhile, the CMG-H electrodes exhibited an obvious bigger integral area compared with others, which implies that CMG-H can release more current under same potential. This is the reason why the CMG-H, which do not possess the strongest LiPSs chemisorption ability, can exhibit the best Li–S cell performance. Moreover, these results indicate that the liquid–solid conversions for LiPSs to Li_2_S can be readily performed in CMG-H, which should be attributed to the abundant generated 
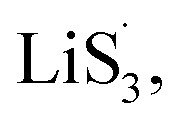
 followed by the disproportionated nucleation reaction. The fastest Li^+^ ion diffusion also confirmed the rapid conversion *via* the Randles–Sevcik equation, which validated our introduced NTR in the peak current vision (see further Discussion in ESI and Fig. S11 and S12†). In addition, the GITT measurements were performed. A constant current density of 167.2 mA g^−1^ (the theoretical 0.1C current for 1 g of sulfur) was applied for 1 h, and a pulse duration of 2.5 h was applied to collect the potential response after the first active cycle, as presented in Fig. 5(e), (f) and S13.†^54^ CMG-H consistently expressed the lowest reaction resistances in both charge/discharge processes, suggesting a minimum LiPS interconversion barrier.

**Table 1:** The reaction equation and electron-transfer number of Li–S batteries

Peak	Reaction	Phase	Electron transfer number	Reaction
1	S_8_ + 4e^−^ + 4Li^+^ → 2Li_2_S_4_	Solid → liquid	4	(1)
2	2Li_2_S_4_ + 12e^−^ + 12Li^+^ → 8Li_2_S	Solid → liquid	12	(2)

## Conclusions

In summary, we introduced sulfur vacancies into heterojunction materials and conducted a systematic investigation of the chemisorption and kinetics of heterojunctions with increasing sulfur vacancies in Li–S cells. Chemisorption can be harmonized to realize a uniform distribution of LiPSs in the heterojunction. The introduction of sulfur vacancies can generate a large amount of 
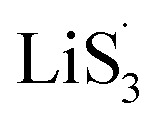
 radicals, which can promote the nucleation of Li_2_S *via* a spontaneous disproportionated reaction. The formation of Li_2_S can participate in the hybrid current in the early discharge stage to reduce the accumulation of liquid LiPSs. Additionally, the defined descriptor nucleation transformation ratio was applied to quantitatively elucidate the kinetic behaviors of the materials and understand Li–S full battery performance. The catalytic mechanisms were therefore elucidated and paved the way for material design and theoretical direction.

## Author contributions

C. L. and Z. W. directed the project. H. Tang and R. Xu performed the main experimental works. Y. Z., F. W. and H. W. participate in some of experimental works. R. X. analysed the data and wrote the manuscript. R. X. and H. T. contributed equally. All the authors discussed the results.

## Conflicts of interest

There are no conflicts to declare.

## Supplementary Material

SC-013-D2SC01353C-s001
