# Supplementary material for: Enhanced catalysis of [image] radical-to-polysulfide interconversion via increased sulfur vacancies in lithium–sulfur batteries
Source: Chem Sci. 2022 May 10;13(21):6224–32. doi: 10.1039/d2sc01353c (PMC9159087; doi:10.1039/d2sc01353c)
Supplement: SC-013-D2SC01353C-s001 [file SC-013-D2SC01353C-s001.pdf]

Electronic Supplementary Material (ESI) for Chemical Science.
This journal is © The Royal Society of Chemistry 2022

Supporting information

Enhanced Catalysis of $LiS_3^{\bullet}$ Radical-to-Polysulfide Interconversion via Increased Sulfur Vacancies in Lithium–Sulfur Batteries

Rui Xu, [†a] Hongan Tang, [†a] Yuanyuan Zhou, [a] Fangzheng Wang, [a] Hongrui Wang, [a] Minhua Shao,[b] Cunpu Li, *[a] Zidong Wei*[a]

[†]The State Key Laboratory of Power Transmission Equipment & System Security and New Technology, Chongqing Key Laboratory of Chemical Process for Clean Energy and Resource Utilization, School of Chemistry and Chemical Engineering, Chongqing University, Shazhengjie 174, Chongqing 400044, China.
[‡]Department of Chemical and Biological Engineering, The Hong Kong University of Science and Technology, Clear Water Bay, Kowloon, Hong Kong

Correspondence to: lcp@cqu.edu.cn; zdwei@cqu.edu.cn

[§] Rui Xu and Hongan Tang contributed equally to this paper.

Further Discussion

To elucidate the kinetics difference of redox peaks, the cyclic voltammograms were studied further (**Fig. 5(a-c)**). The power–law relationships between peak current and the scan rates were analyzed based on the following equation:[1, 2]

$$i = av^b \qquad (1)$$

b is positively and correlate to the corresponding Li^+ diffusion, which can be determined from the slope of the plot of ***log i*** *vs.* ***log υ***. The ***b***-values of the cathodic Peak 1 (high-order LiPSs to Li_2S_4) and Peak 2 (Li_2S deposition) were calculated and listed in **Figure S11**. Along with the increasing of sulfur vacancies, it can be seen that the ***b***-values were 0.53, 0.58, and 0.60 at Peak1 for CMG-L, CMG-M, and CMG-H, respectively. This is consistent with the abundant active sites of CMG-H, which will easily covert element sulfur to Li_2S_4. Besides, CMG-H exhibited the highest ***b***-values at Peak 2, implying that the Li_2S can effectively and uniformly deposit *via* Route II. The liquid-phase $\mathrm{LiS_3^{\bullet}}$ will convert to the rapidly liquid-phase Li_2S_3, subsequently rapidly transforming the solid-phase Li_2S_2 and Li_2S *via* the reduction reaction.

For confirming the improvement of the redox kinetics, the Li^+ ion diffusion properties were evaluated by Randles–Sevcik equation:

$$\boldsymbol{i_p = (2.69 \times 10^5) n^{1.5} A\, D_{Li^+}^{0.5}\, \nu^{0.5}\, C_{Li^+}} \qquad (2)$$

where $\boldsymbol{i_p}$ is the peak current density, ***n*** is the charge transfer number, ***A*** is the area of electrode. $\boldsymbol{D_{Li^+}^{0.5}}$ is the Li^+ ion diffusion coefficient, $\boldsymbol{\nu^{0.5}}$ is the scan rate, and $\boldsymbol{C_{Li^+}}$ is the concentration of Li^+ ions in the cathode. Therefore, the value of $\boldsymbol{i_p/\nu^{0.5}}$ can represent the Li^+ diffusion rate because ***n***, ***A***, and $\boldsymbol{C_{Li^+}}$ are unchanged. Li^+ ion diffusion coefficient was determined by both the conductibility and reaction kinetics with S-species.[3] As shown in **Figure S12**, CMG-H exhibits highest $\boldsymbol{i_p/\nu^{0.5}}$ in both the conversion of Peak 1 and Peak 2. The reaction kinetics were accelerated for the conversions of Peak 1 and Peak 2, especially Li_2S deposition. It was also verified that the solid-phase Li_2S were uniformly deposited from **Figure S12**, attributing to the faster Li^+ ion diffusion. Thus, with the increase of sulfur vacancies, active $\mathrm{LiS_3^{\bullet}}$ will rapidly convert to Li_2S_3, following transform to solid-phase Li_2S_2 and Li_2S. High Li^+ conductibility and fastest reaction kinetics endow the CMG-H the best battery performance.

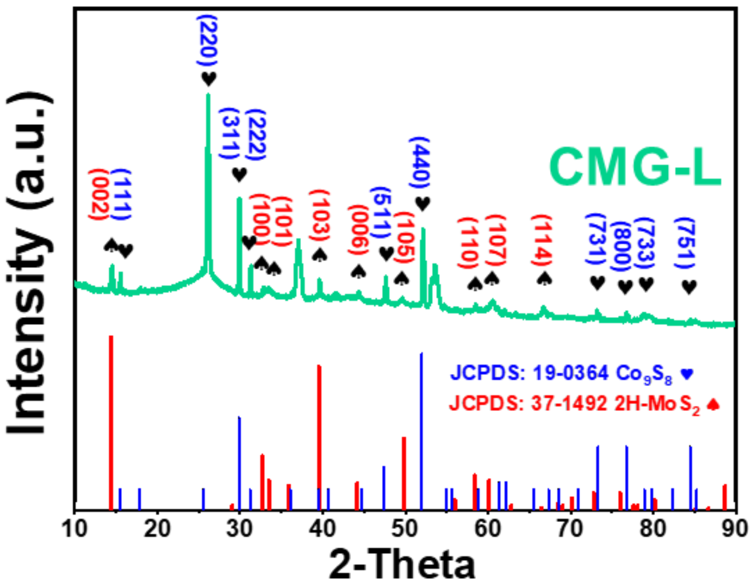

Figure S1. XRD spectrum of CMG-L.

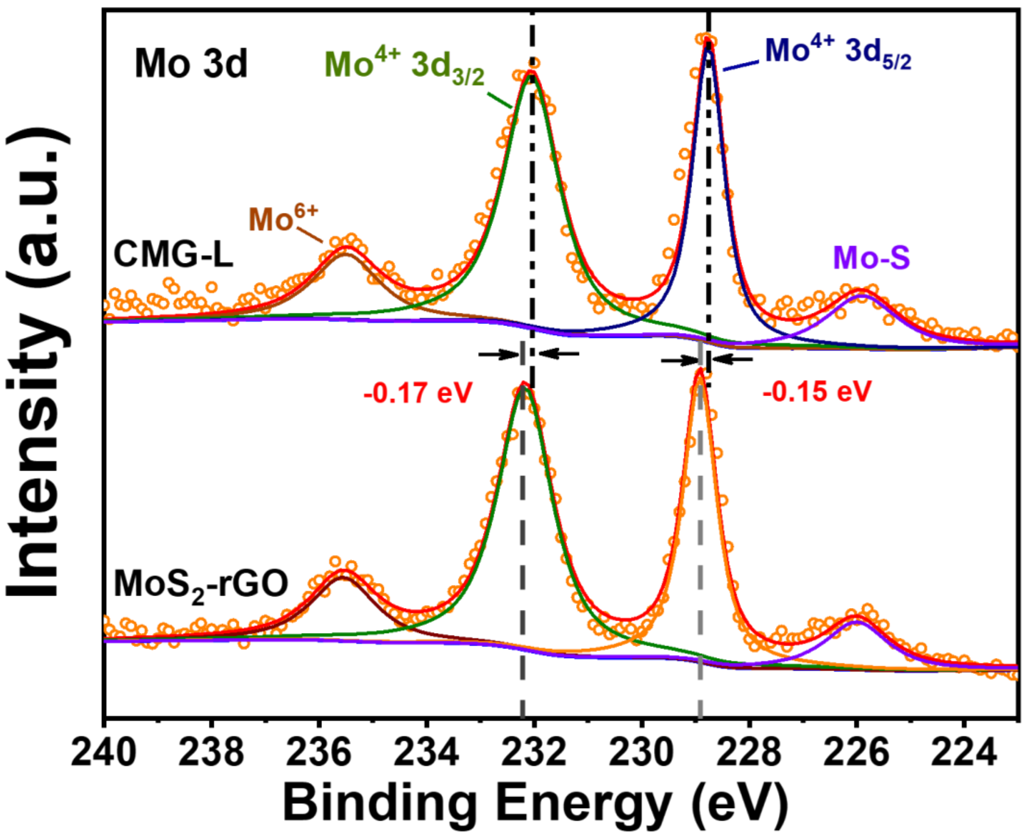

Figure S2. XPS spectrum of the CMG-L and MoS_2-rGO.

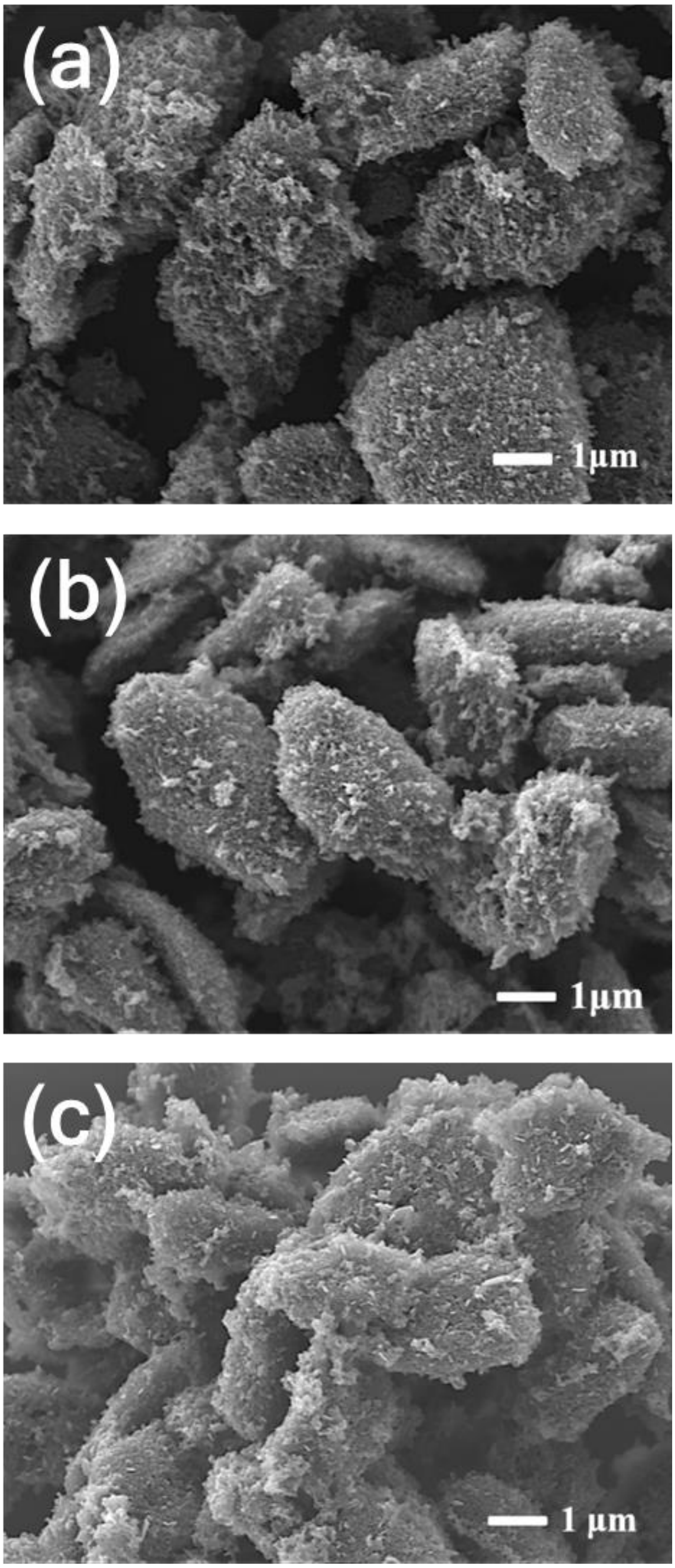

Figure S3. The SEM images of (a) CMG-L (b) CMG-M and (c) CMG-H.

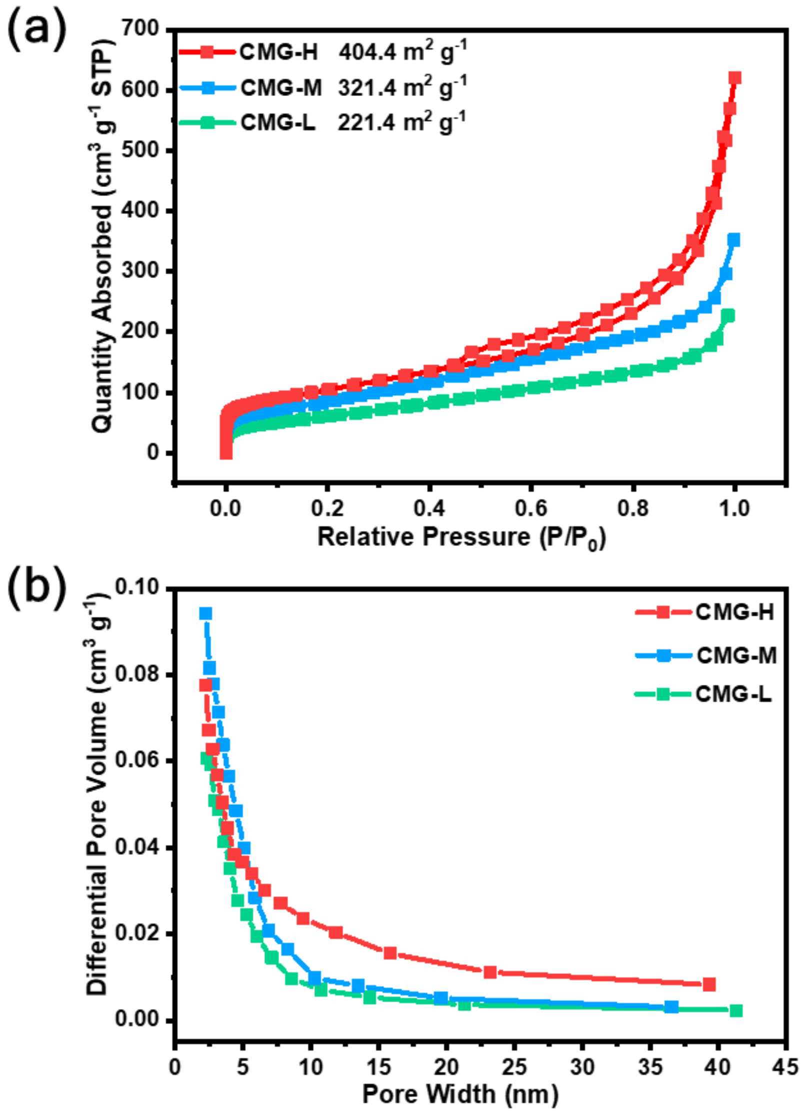

Figure S4. The N_2 adoption-desorption isotherms (a) and pore distribution (b) of CMG-L, CMG-M and CMG-H.

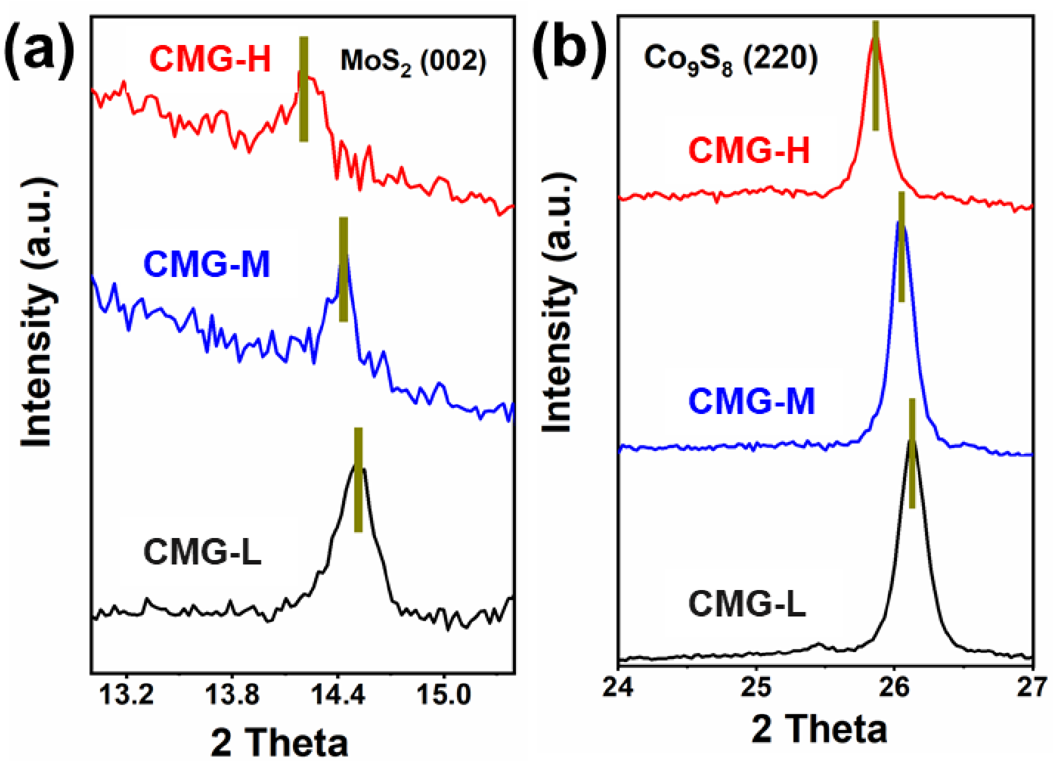

Figure S5. The expanded view of XRD patterns in 2θ regions of CMG-L, CMG-M and CMG-H.

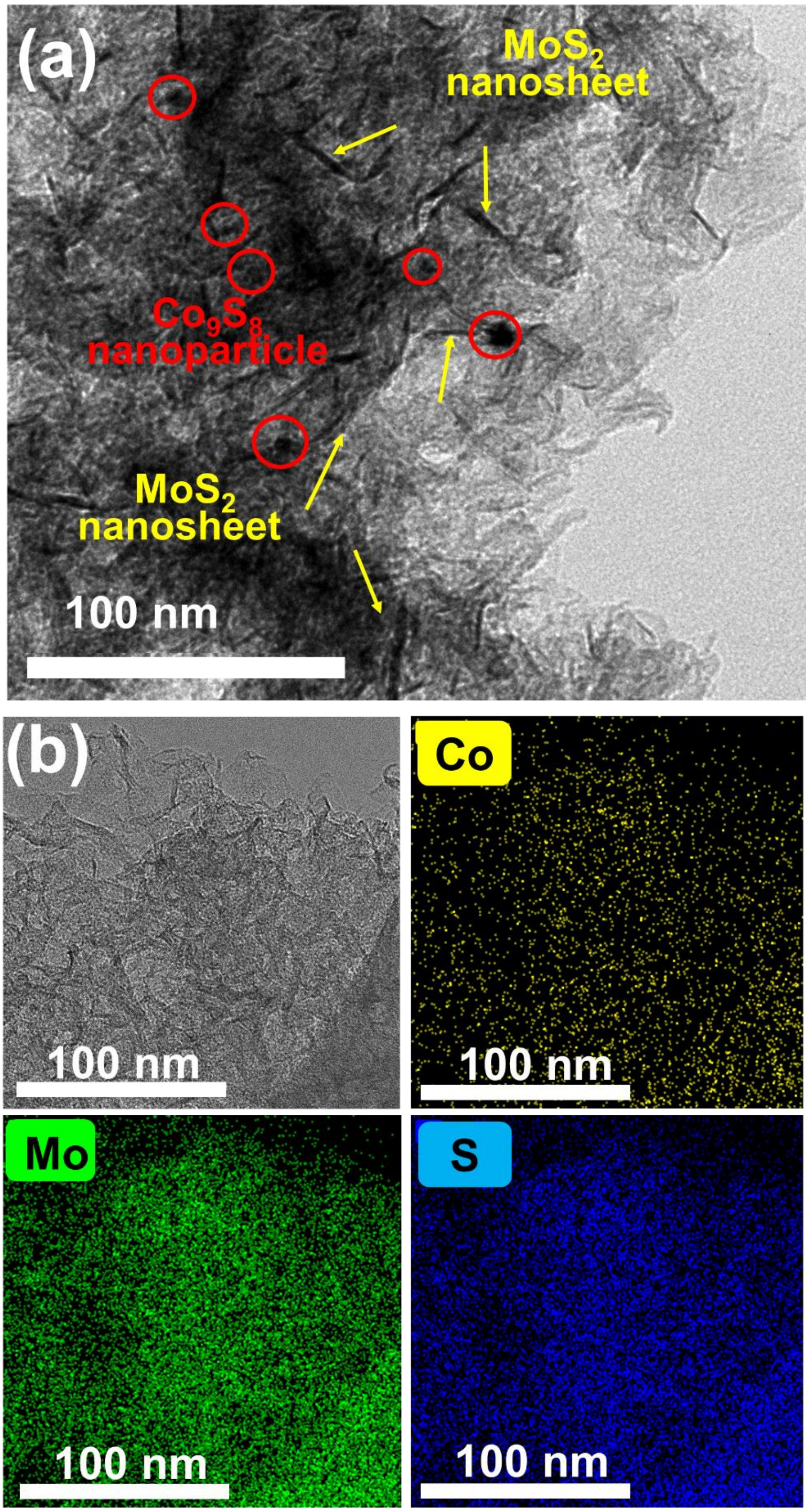

Figure S6. (a) TEM images and (b) elemental mappings images of CMG-H.

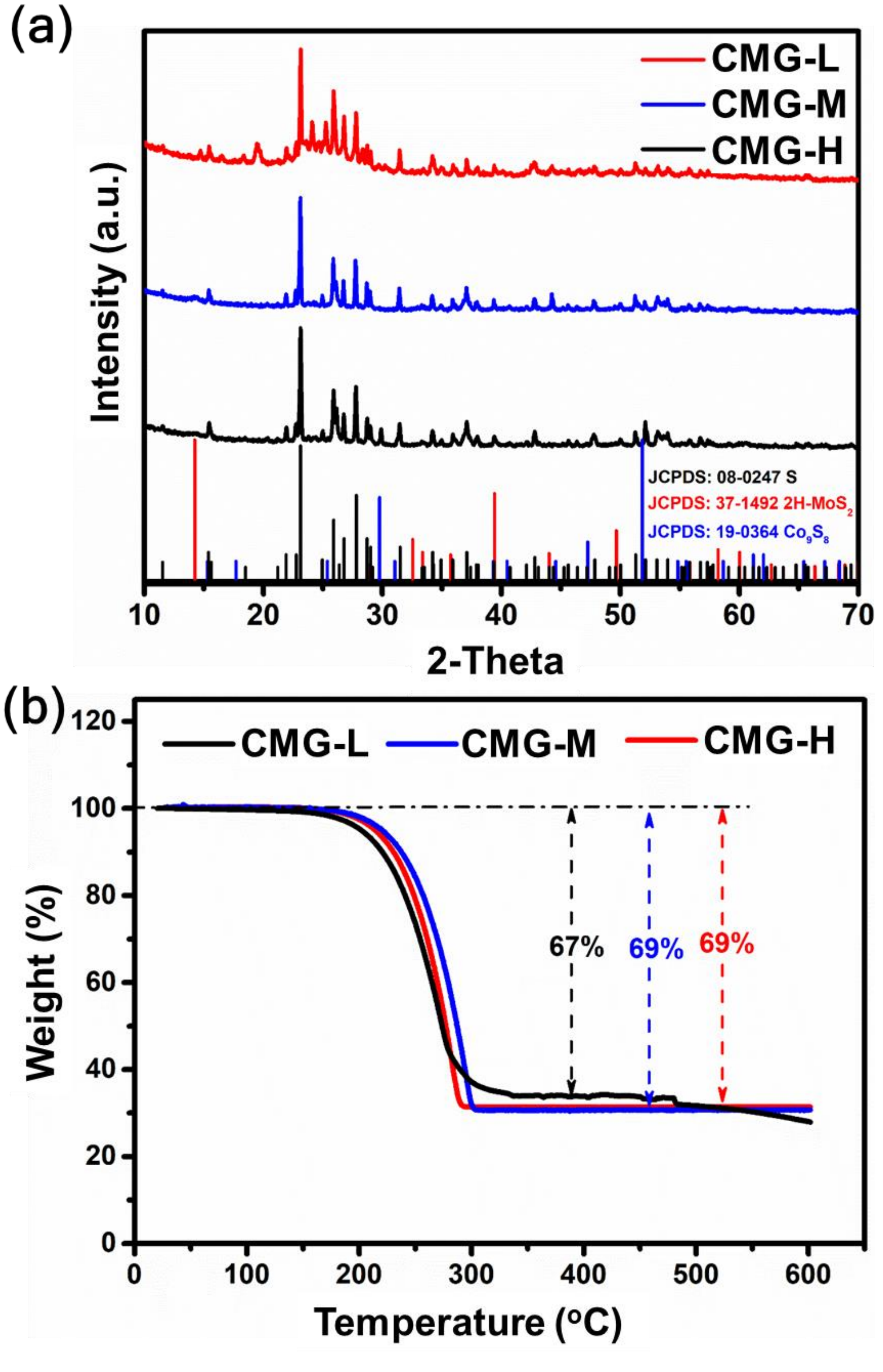

Figure S7. XRD patterns (a) and TG curves (b) of S@CMG-L, S@CMG-M and S@CMG-H.

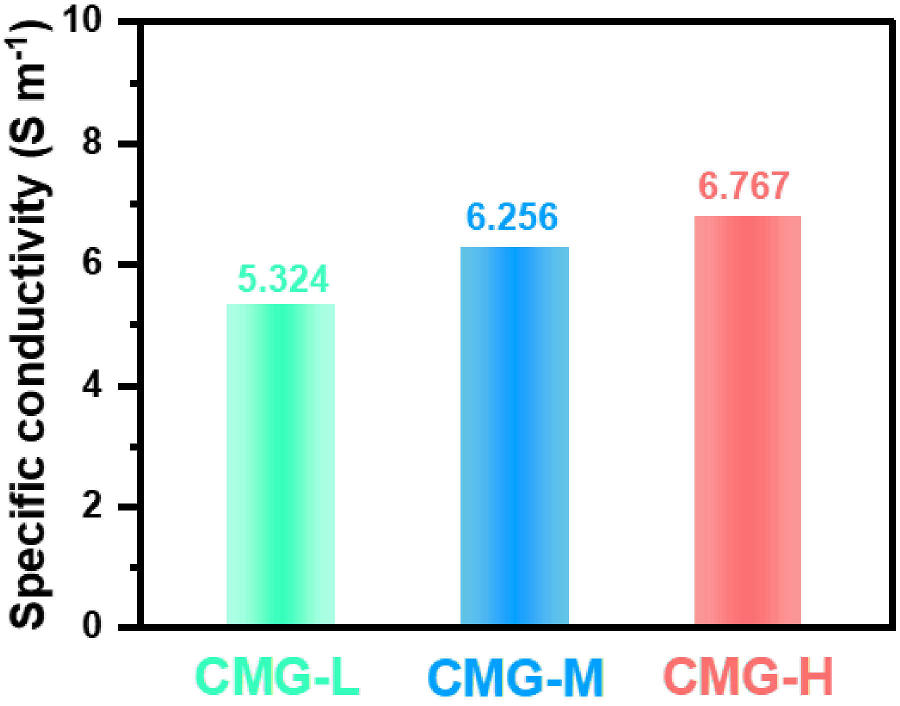

Figure S8. The specific conductivity of CMG materials with different sulfur vacancy.

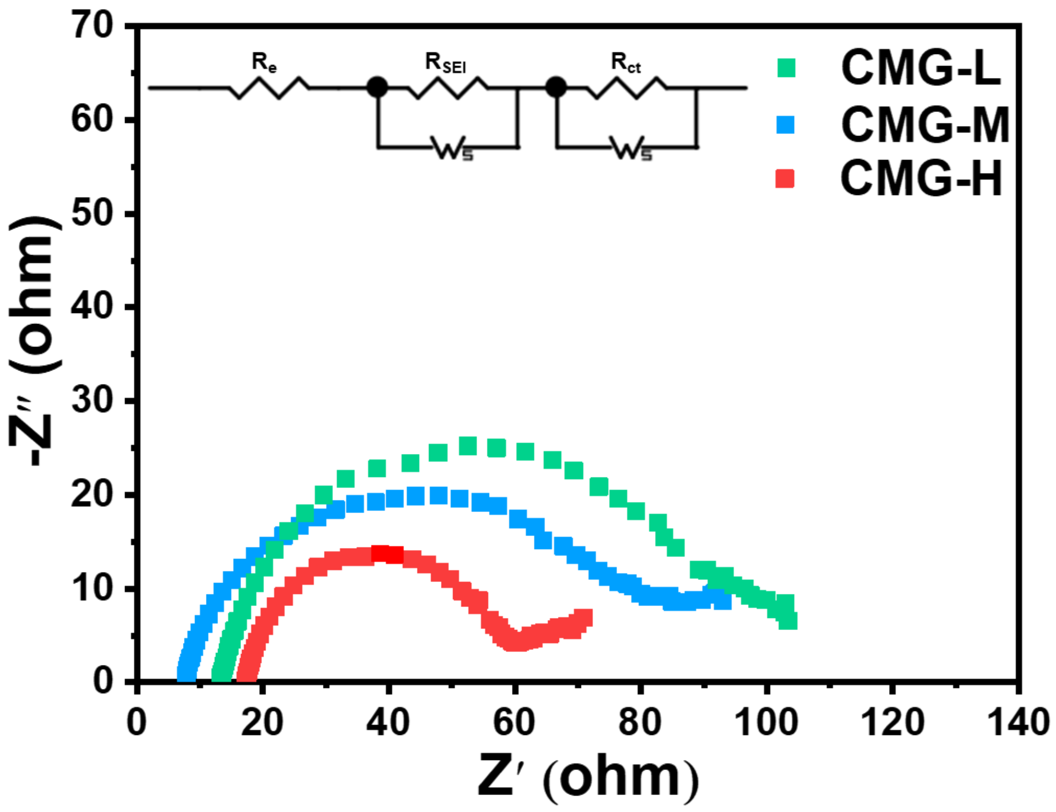

Figure S9. Electrochemical impedance spectra of the coin cells after activation by the galvanostatic charge-discharge test at 0.2 C.

The electron transfer ratio (high-order LiPSs conversions and Li_2S deposition) calculation

The **NTR** can be calculated *via* CV curves. Firstly, we should define the baseline for cathodic peaks of CV curves. Then, the integral area (A_t) of cathodic peaks (peak 2 and peak 1) can be calculated *via* integration between Peak line and baseline. Moreover, the amount of electron transfer per gram (C_e) was achieved *via* A_t (A·V) divided by scan rate (v, V·s^{-1}), excluding the scan rate influence. Finally, the **NTR** can be calculated, which reveals the relationship between the high-order LiPSs conversions and Li_2S deposition.

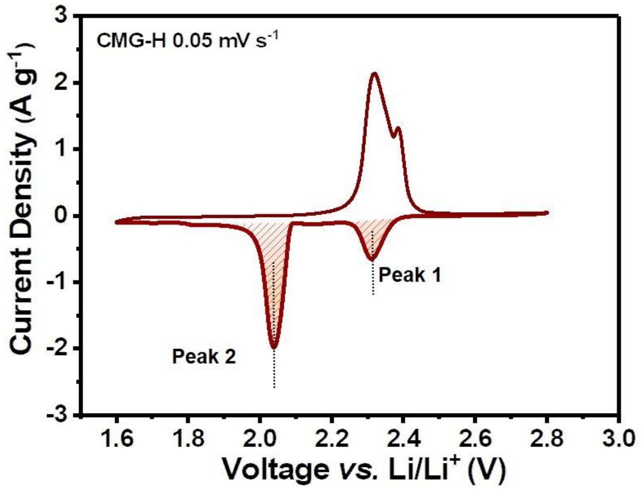

Figure S10. The calculation of integration areas on cathodic peaks.

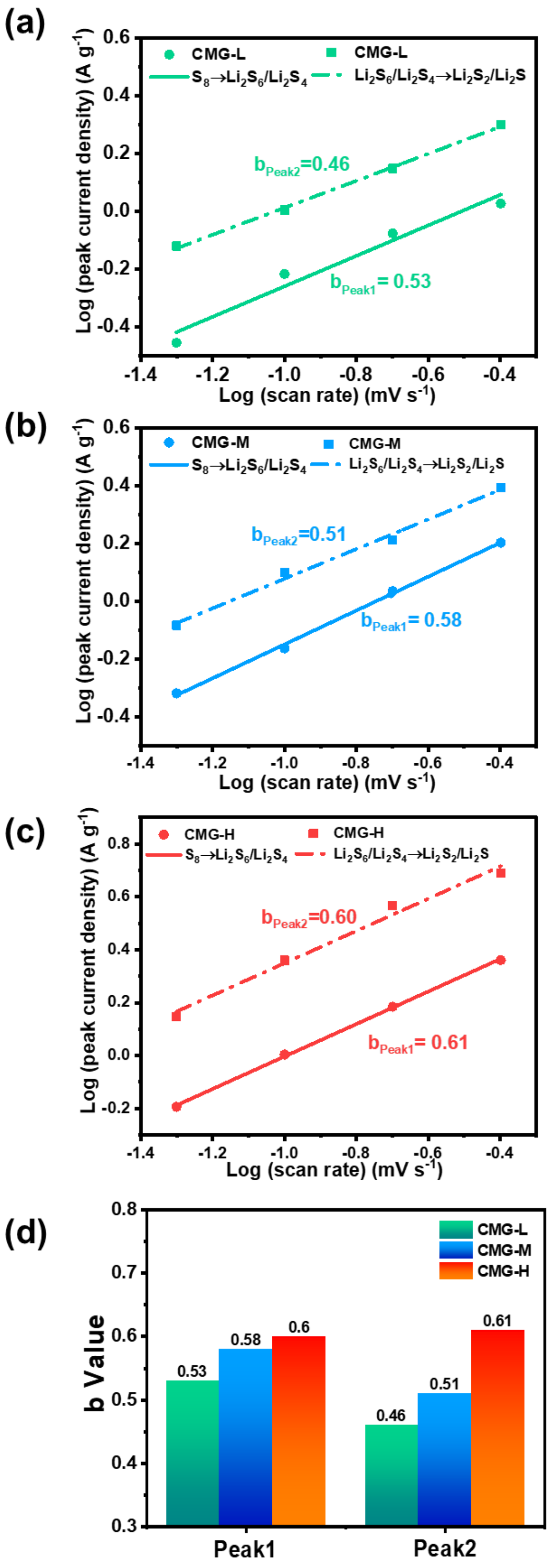

Figure S11. Plots of log(current) *vs.* log(scan rate) in Peak 1, Peak 2 for (a) CMG-L, (b) CMG-M., (c) CMG-H; (d) the b values of CMG-L, CMG-M and CMG-H in Peak 1, Peak 2.

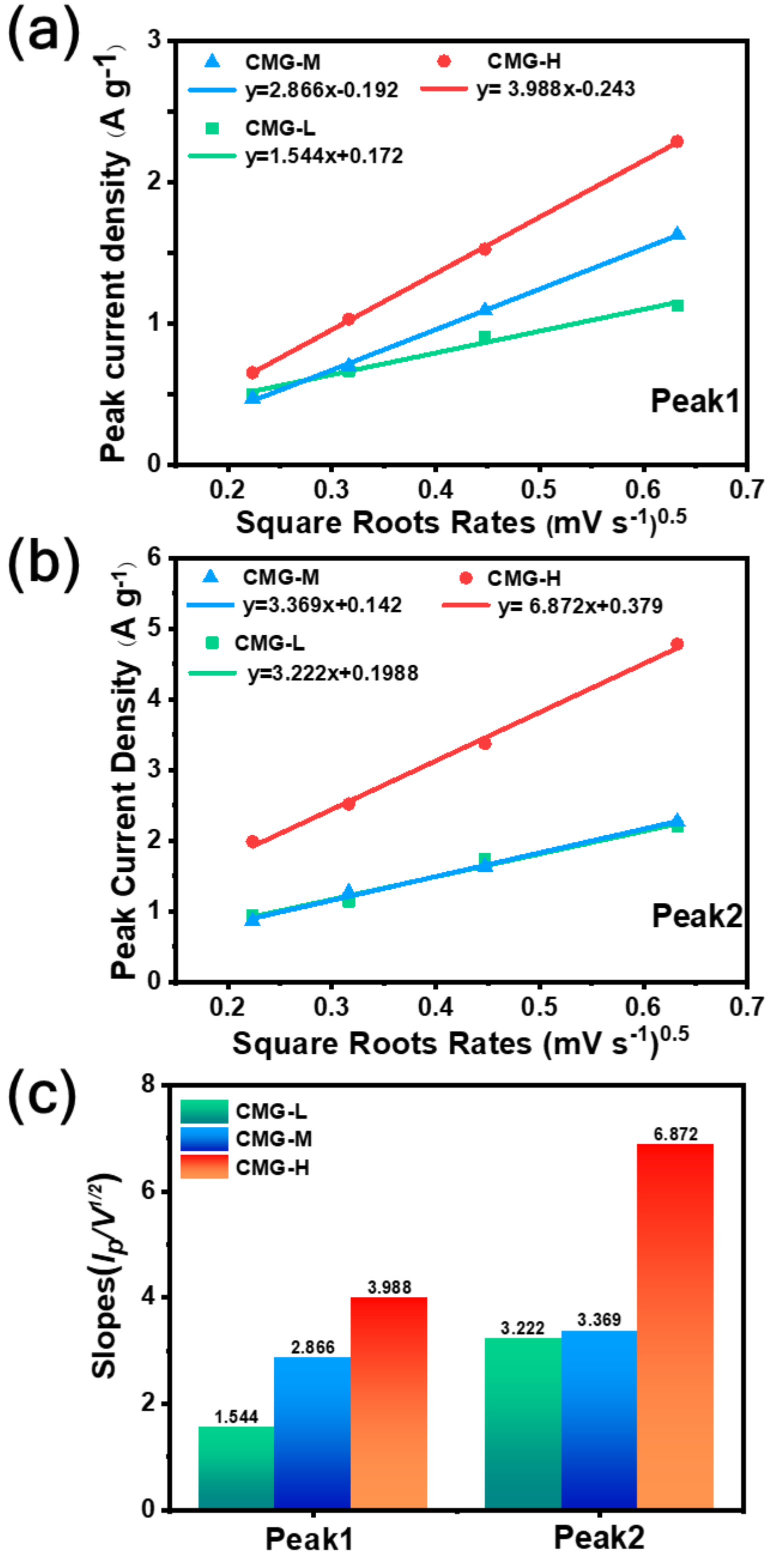

Figure S12. Plots of CV peak current density vs the square root of the scan rates for (a) Peak 1 and (b) Peak 2; (c) the slopes ($i_p/\ v^{0.5}$) values of CMG-L, CMG-M and CMG-H.

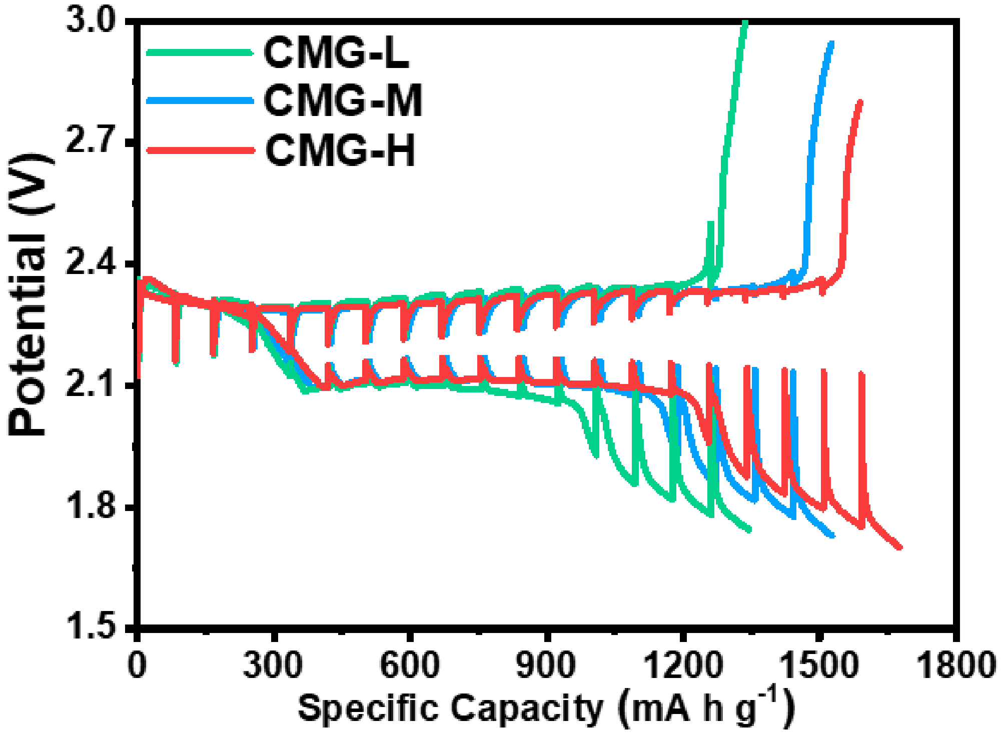

Figure S13. The potential response curves of the three electrodes during GITT measurement.

Table S1. The variety of sulfur defect concentration analyzed by XPS.

Sample	Co-Mo-S (atom ratio)	Co_9S_8/MoS_2(stoichiometric ratio)	Sulfur loss ratio
CMG-L	0.79-1.09-2.9	$Co_{0.79}S_{0.70}/Mo_{1.09}S_{2.18}$	0.12
CMG-M	0.52-0.99-2.35	$Co_{0.52}S_{0.46}/Mo_{0.99}S_{1.98}$	-0.09
CMG-H	0.51-0.86-1.66	$Co_{0.51}S_{0.45}/Mo_{0.86}S_{1.72}$	-0.51

Table S2. The equivalent circuit and fitting resistance of symmetric batteries.

Full cell	R_e	R_{SEI}	R_{ct}	Equivalent circuit
CMG-L	13.19	40.54	61.02	
CMG-M	7.43	36.65	41.09	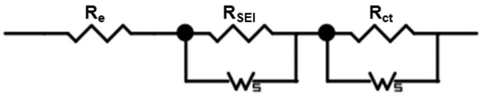
CMG-H	15.59	27.58	30.16	

Table S3. The absolute values of the integration areas (peak 2 and peak 1) and NTR with different scan rates.

Sample	Scan rates (mV/s)	Peak1 ($A\cdot V\cdot g^{-1}$)	Peak2 ($A\cdot V\cdot g^{-1}$)	NTR (peak2 vs. peak1)
CMG-L	0.05	0.02256	0.06246	2.78
	0.1	0.05401	0.14182	2.63
	0.2	0.07913	0.19634	2.48
	0.4	0.12915	0.31063	2.40
CMG-M	0.05	0.02724	0.07996	2.93
	0.1	0.04566	0.12438	2.72
	0.2	0.08488	0.21956	2.58
	0.4	0.15109	0.37987	2.51
CMG-H	0.05	0.03631	0.10746	2.96
	0.1	0.06338	0.18696	2.95
	0.2	0.11984	0.34812	2.91
	0.4	0.20008	0.57968	2.90

References

1 W. G. Lim, C. Jo, A. Cho, J. Hwang, S. Kim, J. W. Han and J. Lee, *Adv.Mater.*, 2019, **31**, e1806547.

2 L. Xu, H. Zhao, M. Sun, B. Huang, J. Wang, J. Xia, N. Li, D. Yin, M. Luo, F. Luo, Y. Du and C. Yan, *Angew. Chem., Int. Ed*, 2019, **58**, 11491-11496.

3 Y. Tao, Y. Wei, Y. Liu, J. Wang, W. Qiao, L. Ling and D. Long, *Energy Environ. Sci.*, 2016, **9**, 3230-3239.
